# Implication of High Body Fat Percentage on Migraine Chronification in Premenopausal Females

**DOI:** 10.1155/2022/8219254

**Published:** 2022-10-15

**Authors:** Pooja Ojha, Varun Malhotra

**Affiliations:** ^1^All India Institute of Medical Sciences, Jodhpur, Rajasthan, India; ^2^Era's Lucknow Medical College, Lucknow, Uttar Pradesh, India

## Abstract

**Background:**

Chronic migraine, being a debilitating headache disorder, needs assessment of the risk factors implicated in its occurrence. We investigated the potential role of obesity as a risk for chronic migraine in premenopausal females with episodic migraine.

**Methods:**

In this analytical study, body fat% was compared between episodic and chronic migraine patient groups. The standard criteria of the international classification of headache disorder were used for the diagnosis. Demographic data, clinical details of migraine, and anthropometric measurements were collected using structured questions and standardized techniques. Pearson's correlation (*r*) was estimated to assess the concordance between body fat% and migraine frequency. High body fat%'s implication on chronic migraine which was adjusted for body mass index (BMI), and the use of oral contraceptives was determined using logistic regression analysis.

**Results:**

A total of 168 premenopausal female migraineurs, with a mean (Standard deviation) age of 33.0 (±9.0) years, were enrolled in the study. BMI and high body fat% were significantly associated with chronic migraine (*p* < 0.05). There was a weak positive, but significant, correlation between body fat% and migraine frequency (*r* = 0.185, *p* < 0.017). The presence of high body fat was found to increase the risk of chronic migraine by 2.8 times (confidence interval 1.4–5.6; *p* < 0.003).

**Conclusion:**

The amount of fat mass in the body relates to the clinical characteristics of migraine. There is an increased risk of developing chronic migraine in patients having high body fat. Weight control measures can be targeted for the prevention of migraine worsening.

## 1. Introduction

Migraine symptomatology and disability are heterogeneous in presentation and vary in combinations [1]. Migraine headache has been found to be influenced by a myriad of predisposing factors, wherein some of them may probably play a role as migraine precipitants [2]. An unattended moderate intensity headache can gradually worsen over time and accentuate in frequency. Reportedly, about 3% of patients with episodic migraine, attack frequency less than 15 per month, can eventually transform into chronic disease in due course of months to years [3]. Reported literature suggests the role of obesity as a risk towards migraine chronification and the need of additional attention towards the treatment of obese patients [4].

Notably, body fat plays a significant role in physiological and pathological mechanisms as it can affect the action of various drugs, enzymes, and chemicals. Association of body mass index (BMI), a prevalent method for estimating obesity, with migraine has been studied in previous research [5, 6]. BMI has been found to have limited potential when used alone as it does not take into account age, gender, and ethnicity, nor does it differentiate between total body fat mass and lean mass [7]. In addition, BMI has been reported to be an inconsistent predictor of morbidity and mortality in a systematic review [8]. Comparatively, body fat percentage (BF%) is an estimate of the percentage of fat in the body composition [9]. BF% has been found to play a significant role in distinguishing the obese individuals from the healthy ones owing to the greater ability to detect lean mass and fat mass as compared to BMI [10]. It has been proposed to provide a better picture of the risks of weight-related disorders. Patients with higher BMI and greater BF have been reported to have worse clinical features.

Research related to migraine and obesity have predominantly studied BMI with limited investigations on BF%, and the existing data on the relationship between body fat percentages and migraine features are scarce [11–13]. Hence, measurement of BF% becomes a feasible option in this scenario. This study was designed to find an association between obesity based on body fat percentages and migraine clinical features. The findings would be implied in preventing the progression of migraine frequency and disability.

## 2. Methods

### 2.1. Ethics Statement and Patient Recruitment

This analytical study recruited a premenopausal female population diagnosed with episodic migraine (EM) and chronic migraine (CM) from the associated hospital's medicine outpatient department. The females who had regular menstrual periods with less than one missed period in the last year were considered premenopausal and included in the study [14]. International criteria for headache disorders, third edition, were used for the diagnosis of the patients by the neurologist [15]. Ethical clearance was taken from the institute's ethical committee, and written informed consent forms were filled by all the patients before the commencement of the study. Those having other types of headache disorder, secondary headaches, and pregnancy were excluded from the study.

### 2.2. Protocol

All patients were subjected to a common protocol, including interview and anthropometric assessment. The frequency of migraine was assessed as the number of attack days per month. A history of intake of oral contraceptive pills (OCP) was inquired from all patients. The response was reported on a dichotomous scale as yes or no.

### 2.3. Anthropometric Assessment

All measurements were taken preferably in the morning hours, in the laboratory of the Department of Physiology. Measurement of the body weight was kept close to 0.1 kg using a digital weighing scale. Weight was taken with the patient wearing light clothing and standing in the center of the weighing machine. Height was measured using a standard stadiometer. Patients were instructed to look in the Frankfurt plane, with buttocks and ankles touching the scale. Reading was taken to the nearest 1.0 cm. Body mass index was calculated using Quetelet's index. Multiple skin fold thicknesses were measured in mm at four sites, using Harpenden skinfold calipers, to determine the fat mass. Sites used were biceps, triceps, subscapular, and abdominal. The thickness of a vertical fold, raised on the anterior of the arm, was measured for biceps thickness. Triceps skinfold thickness was measured as a fold raised over triceps muscle. The site was kept in between the lateral projection of the acromion of the scapula and the inferior edge of the olecranon process of the ulna. The subscapular fold was raised diagonally, in the inferolateral direction below the inferior angle of the scapula. The abdominal fold was picked in the natural cleavage line on the anterior of the abdomen in the inferomedial direction. All measurements were taken on the same side of the body, preferably the nondominant side. The mean of three measurements was taken for each site. A sum of the skin fold thicknesses was used to calculate body density (BD) using the validated, age-specific, and sex-specific Durnin and Womersley equation [16], and Siri equation [17] was used to convert BD to percentage body fat (BF%).

### 2.4. Statistics

The statistical package for social sciences version 23 for Windows was used for all analysis. Counts (percentage) and mean ± standard deviation (SD) represent categorical and continuous data, respectively. The chi-square test and student *t*-test were used wherever needed for comparison of categorical and continuous data, respectively. Pearson's coefficient of correlation (*r*) was used to ascertain the concordance between body fat% and migraine frequency. The risk for chronic migraine in the presence of high body fat was estimated through binomial logistic regression analysis. Age, BMI, and OCP were treated as potential confounders based on some previous reports [18, 19], and their effect was managed using statistical models to look for an independent effect of body fat. For the construction of the model, patients were categorized into two groups based on BF%. Those with <30% body fat were grouped as normal, while others with >30% body fat were in the high BF% group. Patients were characterized into two groups of normal (<22.9) and high BMI (>22.9) using a cut off value of 22.9 according to the consensus reports on Asian Indians [20]. For age-wise comparison of the effect, patients were grouped into 4 categories of age. Age 18–19, 20–29, 30–39, 40–49 years as groups 1, 2, 3, and 4. Statistical significance was kept at *p* < 0.05.

## 3. Result

A total of 168 patients of episodic and chronic migraine having a mean (SD) age of 33.0 (9.0) years were included in the study. One hundred six patients had EM, and 62 patients had CM. The groups did not differ significantly in age (*p* = 0.907), but a significant difference was found in BMI and BF% in the two groups, both being a little higher in CM (Table 1).

In an unadjusted estimate, an increased risk for CM was found in the patients of EM having high body fat percentage (odds ratio 2.8 with a 95% confidence interval between 1.4–5.7; *p* < 0.002). The effect was further confirmed by the logistic regression analysis which revealed that high BF% is an independent risk factor for developing CM in patients of EM, when adjusted for other covariates (Table 2).

**Figure 1:**
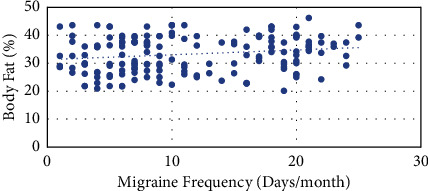
The graph depicts the relation of increasing body fat percentage with increase in migraine frequency.

There was a weak positive but significant correlation between BF% and migraine frequency (*r* = 0.185, *p* < 0.017) (Figure 1).

## 4. Discussion

This study examined the role of obesity in affecting migraine chronicity. Though there was a significant association of BMI and BF% with CM, the latter was found to be an independent predictor of CM in premenopausal patients of EM.

Our study group comprised females in the premenopausal age group. Interestingly, age has been found to affect the clinical manifestation of migraine, and this effect is likely to be demarcated at the menopause [21]. Differential interaction of the adipose tissue and female hormones with increasing age may be a plausible explanation for this observation.

Previously, the relationship between obesity and migraine has been explored but with an emphasis on BMI rather than BF%. In a systematic review, the overweight and obese populations were found to have an overall increased risk of migraine when compared to normal weight individuals [22]. An observation of the consensus reported that the cutoff for BMI, which would correspond to the cutoff for BF% (30% in women), is lower in the Asian Indian population [20]. Nevertheless, the focus gradually shifted to compartmental models for determining obesity, as BMI seemed to be lacking a true estimation of adiposity. We estimated obesity using body fat percentage and found that fat mass had significant bearing in worsening migraine. Several studies have reported findings in agreement with ours. Peterlin et al., in a study estimating migraine prevalence in people with and without abdominal obesity and total body obesity, found that the prevalence varied with the adipose tissue distribution [21]. Rossoni de Oliveira et al. studied the association of anthropometric parameters with migraine features. They were of the opinion that though tenuous but a potential relation exists between obesity and migraine attack frequency, which tends to disappear in the postmenopausal age group [23]. Jahromi and colleagues assessed the effect of fat mass and fat-free mass on migraine. They reported that lower fat-free mass has an adverse effect on migraine occurrence in obese and overweight population [13]. On the other hand, a population-based study reported no difference in obesity among women with active migraine, inactive migraine, and no migraine [24]. Differences may be attributable to the diagnostic and measurement criteria used in these studies. The recruitment of patients with self-reported migraine and measurements can affect the study findings when compared to standard diagnostic criteria and anthropometric assessment. 

The fat depot site has been found to affect the function of the adipocytes, importantly, the amount of secretion of hormones, cytokines, and adiponectin from the adipocytes [25]. Further adding to the cause, the physical activity level has been found to affect the body fat percentage [26] and risk of developing migraine [27]. Stating it together, differences in the ratio of visceral to subcutaneous adipose tissue in the abdominal region in premenopausal and postmenopausal females may be implicated in differences in migraine's clinical manifestation. It is suggested that exercise may have a beneficial effect in desired body fat distribution. Hence, the aforementioned evidence points towards the significance of a weight management program for preventing migraine chronification.

The strengths of this study are a standard diagnostic criterion for patient recruitment and using standardized method for anthropometric measurement, minimizing selection and measurement bias, respectively. Our study included females in the premenopausal age group, as they are more prone to develop migraine. Hence, this study's results cannot be extrapolated to migraine in the menopausal age group or male migraineurs. Next, the regression analysis could eliminate the effect of most potential confounders, but the others less significant have not been addressed. This study has not taken into account male migraineurs who can be potential sufferers. Future studies can be planned with an emphasis on gender-based anthropometric comparison of migraine features. In addition, we have not determined the effects of medication and the status of depression and anxiety on migraine progression that may be considered a limitation of the study.

In conclusion, the premenopausal patients of EM having high BF% may have an increased risk of chronic migraine. Adequate measures need to be taken to facilitate weight control. Also, weight loss can be a potential intervention in preventing migraine chronification.

## Figures and Tables

**Table 1:** Depicts the association of age, BMI, high fat mass, and OCP use with chronic migraine.

Characteristics	Episodic migraine	Chronic migraine	*p*-value
	*n* = 106 mean ± SD or number (%)	*n* = 62 mean ± SD or number (%)	
Age (years)	33.4 ± 8.5	33.2 ± 7.1	0.907
BMI	23.5 ± 4.3	25.0 ± 4.1	**0.030** ^*∗*^
Body fat (%)	32.4 ± 6.5	34.5 ± 5.8	**0.041** ^*∗*^
Taking OCP	46 (43.4)	32 (51.6)	0.303

*n*: number of participants; BMI: body mass index; %: percentage; OCP: oral contraceptive pill; ^*∗*^significant at .05 level with the independent *t*-test.

**Table 2:** The table depicts the logistic regression analysis for likelihood of chronic migraine (*n* = 62), in patients of episodic migraine (*n* = 106) having high body fat percentage, other covariates being adjusted.

Factors	Odds ratio (95% CI)	*p*–value
Age groups (years)		
18–19 versus 40–49	2.6 (0.31–22.8)	0.369
20–29 versus 40–49	1.2 (0.51–2.8)	0.673
30–39 versus 40–49	2.1 (0.9–4.8)	0.069

OCP		
No versus yes	1.4 (0.74–2.7)	0.279

BMI		
Normal versus high	1.2 (0.50–2.4)	0.608
Body fat (%)		
Normal versus high	2.8 (1.4–5.6)	**0.003** ^*∗*^

%: percentage; CI: confidence interval; BMI: body mass index; OCP: oral contraceptive pill; ^*∗*^significant at .05 level.

## Data Availability

Data used to support the findings of this study can be requested from the corresponding author upon reasonable request.

## References

[1] Yalın O. Ö., Uluduz D., Özge A., Sungur M. A., Selekler M., Siva A. (2016). Phenotypic features of chronic migraine. *The Journal of Headache and Pain*.

[2] Vlajinac H., Šipetić S., Džoljić E., Maksimović J., Marinković J., Kostić V. (2003). Some lifestyle habits of female Belgrade university students with migraine and non-migraine primary headache. *The Journal of Headache and Pain*.

[3] Aurora S. K., Kulthia A., Barrodale P. M. (2011). Mechanism of chronic migraine. *Current Pain and Headache Reports*.

[4] Cervoni C., Bond D. S., Seng E. K. (2016). Behavioral weight loss treatments for individuals with migraine and obesity. *Current Pain and Headache Reports*.

[5] Keith S. W., Wang C., Fontaine K. R., Cowan C. D., Allison D. B. (2008). BMI and headache among women: results from 11 epidemiologic datasets. *Obesity*.

[6] Yu S., Liu R., Yang X. (2012). Body mass index and migraine: a survey of the Chinese adult population. *The Journal of Headache and Pain*.

[7] Di Renzo L., Cammarano A., De Lorenzo A. (2018). The missclassification of obesity affects the course of migraine. *The Journal of Headache and Pain*.

[8] Chang S. H., Beason T. S., Hunleth J. M., Colditz G. A. (2012). A systematic review of body fat distribution and mortality in older people. *Maturitas*.

[9] Li L., Wang C., Bao Y., Peng L., Gu H., Jia W. (2012). Optimal body fat percentage cut-offs for obesity in Chinese adults. *Clinical and Experimental Pharmacology and Physiology*.

[10] Goonasegaran A. R., Nabila F. N., Shuhada N. S. (2012). Comparison of the effectiveness of body mass index and body fat percentage in defining body composition. *Singapore Medical Journal*.

[11] Bigal M. E., Liberman J. N., Lipton R. B. (2006). Obesity and migraine: a population study. *Neurology*.

[12] Peterlin B. L., Rapoport A. M., Kurth T. (2010). Migraine and obesity: epidemiology, mechanisms, and implications. *Headache*.

[13] Jahromi S. R., Abolhasani M., Meysamie A., Togha M. (2013). The effect of body fat mass and fat free mass on migraine headache. *Iran Journal of Neurology*.

[14] Martin V. T., Pavlovic J., Fanning K. M., Buse D. C., Reed M. L., Lipton R. B. (2016). Perimenopause and menopause are associated with high frequency headache in women with migraine: results of the American migraine prevalence and prevention study. *Headache: The Journal of Head and Face Pain*.

[15] Headache Classification Committee of the International Headache Society IHS (2013). The international classification of headache disorders, 3rd edition (beta version). *Cephalalgia*.

[16] Durnin J. V. G. A., Womersley J. (1974). Body fat assessed from total body density and its estimation from skinfold thickness: measurements on 481 men and women aged from 16 to 72 Years. *British Journal of Nutrition*.

[17] Siri W. E. (1961). Body composition from fluid spaces and density: analysis of methods. *Techniques for Measuring Body Composition*.

[18] Kristoffersen E. S., Børte S., Hagen K., Zwart J. A., Winsvold B. S. (2020). Migraine, obesity and body fat distribution–a population-based study. *The Journal of Headache and Pain*.

[19] Edlow A. G., Bartz D. (2010). Hormonal contraceptive options for women with headache: a review of the evidence. *Reviews in Obstetrics and Gynecology*.

[20] Misra A., Chowbey P., Makkar B. M. (2009). Consensus statement for diagnosis of obesity, abdominal obesity and the metabolic syndrome for Asian Indians and recommendations for physical activity, medical and surgical management. *Journal of the Association of Physicians of India*.

[21] Peterlin B. L., Rosso A. L., Rapoport A. M., Scher A. I. (2010). Obesity and migraine: the effect of age, gender and adipose tissue distribution. *Headache: The Journal of Head and Face Pain*.

[22] Ornello R., Ripa P., Pistoia F. (2015). Migraine and body mass index categories: a systematic review and meta-analysis of observational studies. *The Journal of Headache and Pain*.

[23] Rossoni de Oliveira V., Camboim Rockett F., Castro K., da Silveira Perla A., Chaves M. L., Schweigert Perry I. D. (2013). Body mass index, abdominal obesity, body fat and migraine features in women. *Nutricion Hospitalaria*.

[24] Mattsson P. (2007). Migraine headache and obesity in women aged 40–74 years: a population-based study. *Cephalalgia*.

[25] Kissebah A. H., Krakower G. R. (1994). Regional adiposity and morbidity. *Physiological Reviews*.

[26] Bradbury K. E., Guo W., Cairns B. J., Armstrong M. E. G., Key T. J (2017). Association between physical activity and body fat percentage, with adjustment for BMI: a large cross-sectional analysis of UK Biobank. *BMJ Open*.

[27] Milde-Busch A., Blaschek A., Borggrafe I., Heinen F., Straube A., von Kries R. (2010). Associations of diet and lifestyle with headache in high-school students: results from a cross-sectional study. *Headache: The Journal of Head and Face Pain*.

